# Mitochondrial complex I inhibition enhances astrocyte responsiveness to pro-inflammatory stimuli

**DOI:** 10.1038/s41598-024-78434-y

**Published:** 2024-11-08

**Authors:** Lena Wischhof, Amal John Mathew, Lorenzo Bonaguro, Marc Beyer, Dan Ehninger, Pierluigi Nicotera, Daniele Bano

**Affiliations:** 1 German Center for Neurodegenerative Diseases (DZNE), Venusberg-Campus 1, Gebäude 99, 53127 Bonn, Germany; 2 PRECISE Platform for Single Cell Genomics and Epigenomics, DZNE and University of Bonn and West German Genome Center, Bonn, Germany; 3 Genomics and Immunoregulation, LIMES Institute, University of Bonn, Bonn, Germany

**Keywords:** Reactive astrocytes, ATP-dependent chromatin remodeling SWI/SNF/BAF complex, Mitochondria, Astrocyte, Mechanisms of disease, Mitochondria

## Abstract

Inhibition of the mitochondrial oxidative phosphorylation (OXPHOS) system can lead to metabolic disorders and neurodegenerative diseases. In primary mitochondrial disorders, reactive astrocytes often accompany neuronal degeneration and may contribute to neurotoxic inflammatory cascades that elicit brain lesions. The influence of mitochondria to astrocyte reactivity as well as the underlying molecular mechanisms remain elusive. Here we report that mitochondrial Complex I dysfunction promotes neural progenitor cell differentiation into astrocytes that are more responsive to neuroinflammatory stimuli. We show that the SWItch/Sucrose Non-Fermentable (SWI/SNF/BAF) chromatin remodeling complex takes part in the epigenetic regulation of astrocyte responsiveness, since its pharmacological inhibition abrogates the expression of inflammatory genes. Furthermore, we demonstrate that Complex I deficient human iPSC-derived astrocytes negatively influence neuronal physiology upon cytokine stimulation. Together, our data describe the SWI/SNF/BAF complex as a sensor of altered mitochondrial OXPHOS and a downstream epigenetic regulator of astrocyte-mediated neuroinflammation.

**Supplementary Information:**

The online version contains supplementary material available at 10.1038/s41598-024-78434-y.

## Introduction

The human brain is a highly complex and heterogenous organ consisting of a variety of cell types with remarkably variable features and biological roles. Although the composition and the total number of cells can vary across brain regions, it is generally assumed that resident non-neuronal cells are more abundant than neurons^1,2^. Among the glial cells, astrocytes are responsible to maintain a wide range of homeostatic functions, such as tissue defense and repair, synaptic plasticity and neurotransmission, extracellular ion buffering and neurotrophic paracrine signaling along with others^3–6^. By physically interacting with endothelial cells and pericytes, astrocytes modulate the blood-brain barrier and the blood flow^7,8^, thereby influencing the availability of nutrients and oxygen to the central nervous system (CNS). Having direct access to biomolecules, astrocytes uptake carbon substrates that are used to support their biosynthetic and energetic demands^9,10^. Moreover, astrocytes can actively store energy reserves in the form of glycogen and lipids that can be eventually mobilized and transferred to neurons as glucose, glycolytic intermediates or other small molecules (e.g., amino acids)^11^. It is known that astrocytes significantly contribute to the three-dimensional structure of the synaptic cleft as well as the spatiotemporal resolution of neurotransmission^6,8,12^. Besides that, an emerging literature suggests an activity-dependent metabolic coupling between neurons and astrocytes, which seems to contribute to mechanisms underlying cognitive function, but also to promote cell survival in response to excitotoxic injuries and stress^3,10,13,14^. Although more recent deep-phenotyping studies provide a better overview of the metabolic needs of the various cell types across brain areas^2,15–17^, it is generally accepted that neurons are more sensitive to aberrant mitochondrial bioenergetics, whereas glia show a lower dependency on mitochondrial OXPHOS to meet their energy demands and biosynthetic needs^3,18^. In this regard, astrocytes tend to have a less efficient electron transport chain (ETC) because individual respiratory complexes are not incorporated in mitochondrial supercomplexes, but rather they remain relatively uncoupled^19^. Although mature astrocytes are considered to be highly glycolytic, it has been suggested that increased mitochondrial biogenesis promotes the maturation of developing astrocytes, with a transient metabolic shift toward mitochondrial OXPHOS as the main biosynthetic source of ATP^20^. Consistent with a key role in astrocyte development, genetic inhibition of mitochondrial biogenesis results in the proliferation of immature astrocytes, which exhibit morphological defects and reduced propensity to support neurite outgrowth and neuronal connectivity^20^. Together, these data suggest that developing astrocytes undergo metabolic reprogramming that tightly depend on a highly functional mitochondrial OXPHOS system. While the metabolic flexibility of immature and mature astrocytes has become evident and mechanistically described by an emerging literature^3,10,11,13,14,18^, it remains uncertain whether mitochondrial defects can influence astrocyte responsiveness to inflammatory insults and, if so, through which transcriptionally regulated epigenetic mechanisms.

Mitochondrial defects have been associated to aging and idiopathic neurodegenerative diseases, including amyotrophic lateral sclerosis (ALS), Parkinson’s and Alzheimer’s disease^21–31^. Furthermore, inherited lesions of either the nuclear or the mitochondrial genome can negatively affect mitochondrial bioenergetics, thereby leading to metabolic disorders with variable ages of onset and clinical manifestations^32–34^. As a consequence of mitochondrial dysfunction, patients may develop neurological symptoms due to neurodegeneration in various tissues of the central and peripheral nervous system^32,34^. In the affected areas, histopathology examinations often reveal clear neuroinflammatory signatures, including gliosis and gliotic scars in proximity of the necrotizing lesions^35,36^. The presence of diffuse gliosis in the CNS may be a secondary response to an injury, however new data in transgenic mouse models suggest that mitochondrial deficient glia may contribute to neuronal death. In constitutive knockout mice, loss of Complex I subunit NDUFS4 causes neuronal degeneration and premature mortality^37,38^, which are partially ameliorated by pharmacological depletion of microglia^39^. However, astrocyte-specific *Ndufs4* knockout in adult mice does not cause obvious neurological phenotypes^40^. Thus, since the role of astrocytes in mitochondrial diseases remains to be better elucidated, it would be of interest to determine if aberrant mitochondrial bioenergetics may promote astrocyte reactiveness and neurotoxicity as an upstream cause, rather than a consequence, during neurodegenerative processes^4,5,41^. Furthermore, there is a considerable interest to identify molecules that may modulate astrocyte proliferation and reactivity, with potential benefits in neurodegenerative conditions. In this complex scenario, we herein report that genetic lesions of the ETC stimulate gliogenesis. Through RNA sequencing and subsequent in silico and in vitro analyses, we found that the activity of the SWI/SNF/BAF complex is required to support the responsiveness of astrocytes to pro-inflammatory stimuli. Together, these findings highlight a new epigenetically controlled response of astrocytes to neuroinflammatory insults, which is amplified when the mitochondrial OXPHOS system is inhibited.

## Results

### Mitochondrial OXPHOS inhibition stimulates gliogenesis in a SWI/SNF/BAF complex-dependent manner

**Fig. 1:**
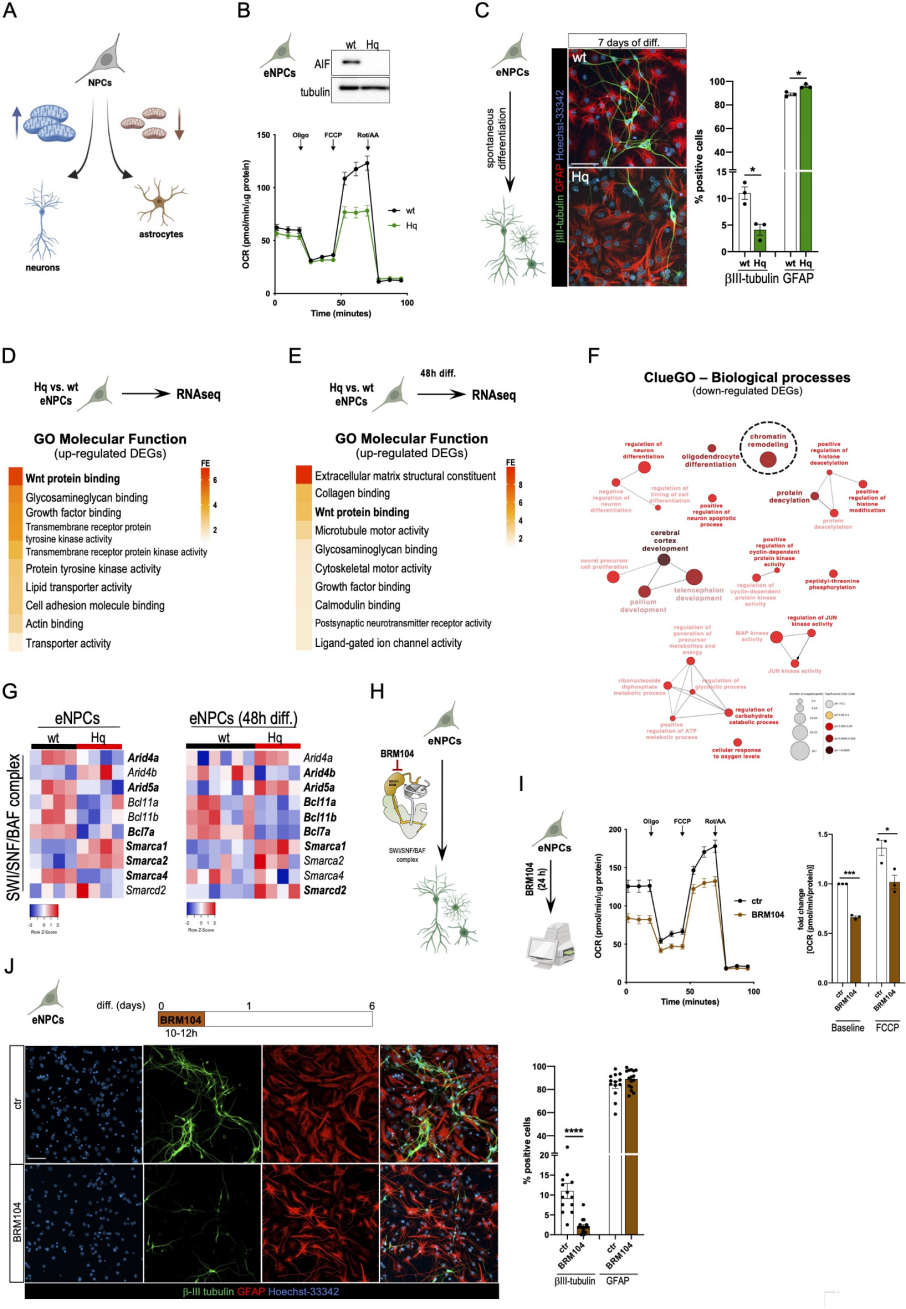
Aberrant mitochondrial OXPHOS alters Wnt signaling and chromatin remodeling. (**A**) Schematic representation of NPC differentiation. Mitochondrial bioenergetics determine whether NPCs become neurons or astrocytes. (**B**) Representative cropped immunoblot of AIF expression levels (top panel) and OCR measurements (bottom panel) in wt and Hq eNPCs (*n* = 2, 5 technical replicates/experiment). (**C**) Representative immunofluorescence images of wt and Hq eNPCs following 7 days of spontaneous differentiation (scale bar = 50 μm). Quantifications of βIII-tubulin- and GFAP-positive cells is shown on the right (two-tailed Student’s *t* test, *p < 0.05; *n* = 3). (**D**-**E**) GO terms of the top dysregulated Molecular Functions for upregulated genes in (**D**) proliferating and (**E**) 48 h-differentiated Hq eNPCs. (**F**) Simplified ClueGO pathway analysis of downregulated genes in proliferating Hq eNPCs. (**G**) Heatmap of differentially expressed SWI/SNF/BAF complex subunits in proliferating (left panel) and 48 h-differentiating (right panel) Hq eNPCs. Significantly dysregulated genes are written in bold. (**H**) Schematic representation of spontaneous eNPC differentiation upon BRM104 treatment. (**I**) Seahorse experiment using wt eNPCs treated with the SW/SNF/BAF complex inhibitor BRM104 (24 h, 2 µM) (two-tailed Student’s *t* test, *p* < 0.05, ***p < 0.001; *n* = 3). (**J**) Immunofluorescence staining of spontaneously differentiated wt eNPCs upon transient treatment (10–12 h) with DMSO (as control; ctr) or 2 µM BRM104 (scale bar = 50 μm). Differentiation was induced by changing the medium after the chemical treatment. Quantification of βIII-tubulin^+^ (neurons) and GAFP^+^ (astrocytes) cells in the resulting cultures is shown on the right (Mann-Whitney test, ****p < 0.0001; *n* = 2, 5–8 technical replicates/condition).

Several lines of evidence indicate that OXPHOS inhibition skews neural progenitor cell (NPC) differentiation toward astrocytes rather than neurons^42–44^ (Fig. 1A). To investigate the underlying molecular mechanisms, we initially adopted mouse-derived embryonic neural progenitor cells (eNPCs) in which the expression of the apoptosis-inducing factor (AIFM1 or AIF) was genetically downregulated. AIF is a component of the mitochondrial disulfide system and, through its functional interaction with CHCHD4/MIA40, promotes the proper ETC biogenesis and mitochondrial OXPHOS^45–49^. Consistent with our previous finding^47,50–52^, partial loss of AIF (~ 80% downregulation) as a consequence of the Harlequin (Hq) mutation caused Complex I defects and inhibited mitochondrial respiration (Fig. 1B). We subjected eNPCs to an in vitro spontaneous differentiation protocol for 7 days and found that Hq mutant eNPC cultures had less cells that were positive for the neuronal marker βIII-tubulin (Fig. 1C). This effect was further associated with a significantly higher percentage of cells positive for the astrocytic marker GFAP (Fig. 1C). We performed RNA-seq analysis in proliferating (i.e., undifferentiated) as well as 48 h-differentiating wt and Hq mutant eNPCs. A total of 987 significantly down- and 1331 upregulated genes were identified in proliferating Hq eNPCs (Supplementary table S1; adjusted *p*-value < 0.05). After 48 h of differentiation, 1189 genes were down- and 2018 genes were up-regulated in Hq mutant cells (Supplementary table S2; adjusted *p*-value < 0.05). For both time points (0 and 48 h of differentiation), Wnt signaling was amongst the top dysregulated pathways (GO Molecular Function; Fig. 1D-E). Additionally, ClueGO enrichment analysis revealed that several of the significantly downregulated genes in Hq eNPCs were linked to neuronal as well as oligodendrocyte differentiation (Fig. 1F), further underscoring the importance of mitochondrial function in cell fate decision. Interestingly, ClueGO also identified a significant enrichment of genes related to chromatin remodeling (Fig. 1F).

We and others showed that the SWI/SNF/BAF complex regulates NPC differentiation and determines cell lineage commitment^53–55^. Building up on our previous studies^53,56^ and taking in consideration these new unbiased ClueGo predictions (Fig. 1F), we sought to focus on the SWI/SNF/BAF complex as a likely epigenetic regulator of astrogenesis. We re-assessed our RNA-seq datasets and found that several SWI/SNF/BAF complex subunits were differentially expressed in both proliferating and 48 h-differentiating Hq eNPCs (Fig. 1G). Consistent with our previous evidence^53^, these data suggest that the SWI/SNF/BAF complex may sense defects of the mitochondrial OXPHOS system and, as a consequence, stimulates gliogenesis rather than neurogenesis. To test whether pharmacological inhibition of the SWI/SNF/BAF complex could alter NPC differentiation (Fig. 1H), we exposed eNPCs to BRM104, a selective chemical inhibitor of the ATPase subunits SMARCA4/BRG1 and SMARCA2/BRM^57^. We found that BRM104-treated cells respired less compared to controls (Fig. 1I). Importantly, brief exposure (10–12 h) of eNPC to BRM104 caused a significant reduction of eNPC-derived βIII-positive cells in spontaneously differentiated cultures (Fig. 1J). Together, these data suggest that the SWI/SNF/BAF complex regulates mitochondrial OXPHOS and, therefore, phenocopies the effect of AIF deficiency during NPC differentiation.

### SWI/SNF/BAF complex inhibition attenuates astrocyte reactivity

**Fig. 2:**
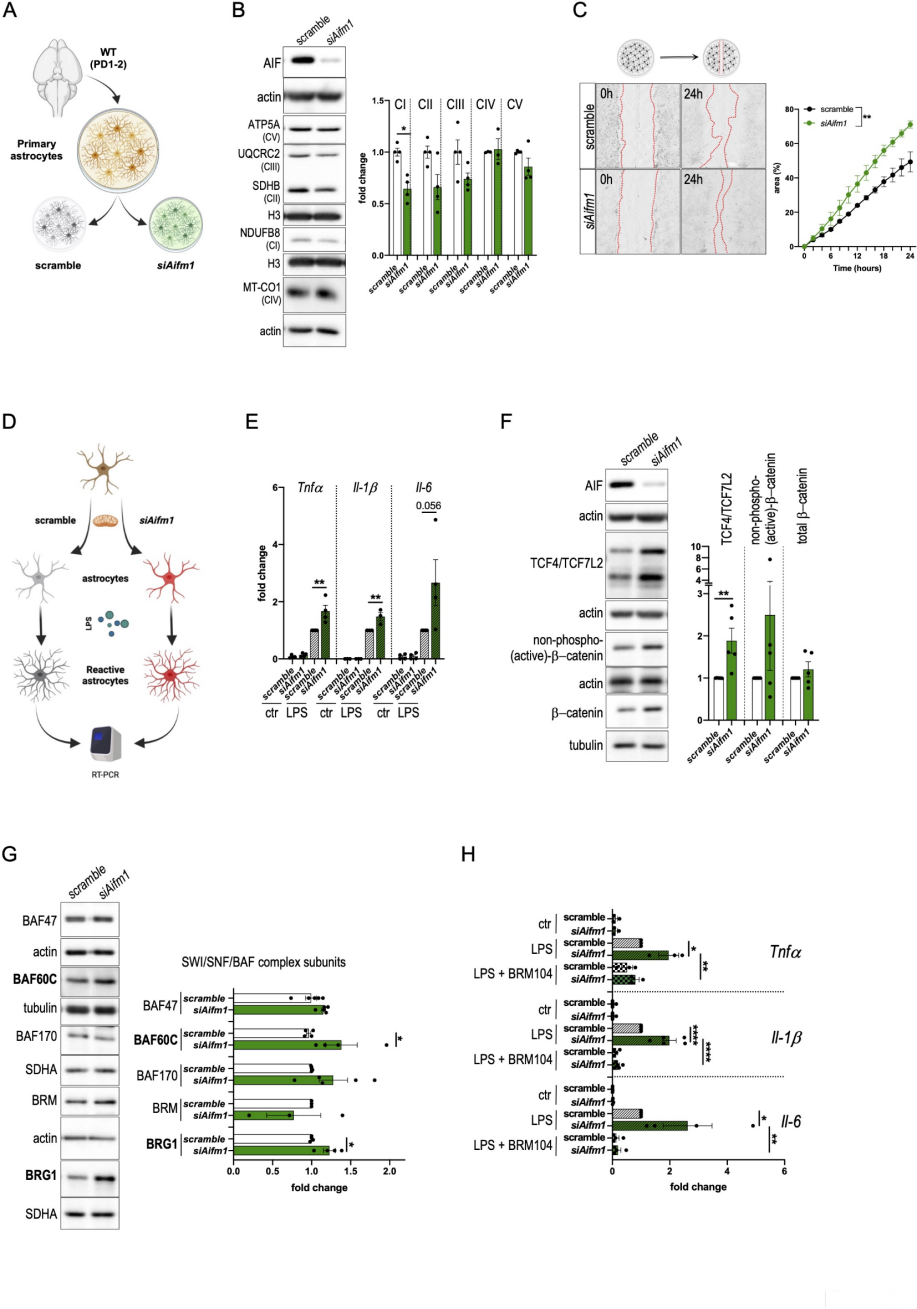
SWI/SNF/BAF complex inhibition attenuates astrocyte reactivity. (**A**) Schematic representation of transient siRNA transfection in mouse-derived astrocytes (postnatal day 1–2). (**B**) siRNA-mediated knockdown of AIF (*siAifm1*) in primary astrocytes isolated from wt pups (PD 1–2). Representative cropped immunoblots of mitochondrial OXPHOS system subunits in scramble and *siAifm1* transfected astrocytes. Densitometries are shown on the right (Mann-Whitney test, *p < 0.05; *n* = 3–4). (**C**) In vitro scratch wound assay using scramble and *siAifm1* transfected astrocytes. Red-dotted lines outline the wound area at 0 h and 24 h post injury. Quantification of wound area closure over time is shown on the right (two-way RM ANOVA followed by Bonferroni’s *post hoc* test for multiple comparisons, **p < 0.01; *n* = 4). (**D**) Schematic representation of the experimental paradigm. Scramble and *siAifm1* transfected astrocytes were exposed to LPS (100 ng/ml, 24 h) or DMSO and mRNA levels assessed by RT-PCR. (**E**) RT-PCR analysis of inflammatory cytokines in scramble and *siAifm1* transfected astrocytes following stimulation with LPS or DMSO (ctr) (one-way ANOVA with subsequent Tuckey’s *post hoc* test for multiple comparisons, **p < 0.01; *n* = 3–4). (**F**) Representative cropped immunoblots for Wnt signaling components in scramble and *siAifm1-* transfected primary astrocytes. Respective densitometries are shown on the right (Mann-Whitney test, **p < 0.01; *n* = 5–7). (**G**) Representative cropped immunoblots of selected SWI/SNF/BAF complex subunits in scramble and *siAifm1-* transfected primary astrocytes. Densitometries are shown on the right (Mann-Whitney test, *p < 0.05; *n* = 4–5). (**H**) RT-PCR analysis of inflammatory cytokine expression in scramble and *siAifm1* transfected astrocytes following LPS stimulation (24 h, 100 ng/ml) in presence or absence of the SWI/SNF/BAF complex inhibitor BRM104 (2 µM). DMSO was used as control (ctr) treatment (one-way ANOVA followed by Bonferroni’s or Tukey’s *post hoc* test for multiple comparisons, *p < 0.05, **p < 0.01, ****p < 0.0001; *n* = 3–5).

To test whether transient inhibition of the mitochondrial OXPHOS enhances astrocyte reactivity, we employed mouse-derived astrocytes that were cultured in vitro and then transfected with small interfering RNA against *Aifm1* to alter Complex I biogenesis (Fig. 2A-B). We performed an in vitro scratch wound assay by mechanically injuring confluent layers of scramble or *siAifm1*-transfected astrocytes. Live-cell imaging (24 h) showed that AIF deficient astrocytes had a higher migration speed and covered the scratched area more rapidly than controls (Fig. 2C). Additionally, we assessed the expression of inflammatory cytokines that were triggered by exposing astrocytes to lipopolysaccharide (LPS, 100 ng/ml) as in previous studies^58–61^ (Fig. 2D). We found that mRNA levels of *Tnfα*,* Il-1β* and *Il-6* were induced upon LPS stimulation, although it was significantly more pronounced in AIF deficient astrocytes compared to controls (Fig. 2E). Together, these data suggest that defective mitochondrial bioenergetics may promote a higher inflammatory-reactive state in astrocytes.

Since mitochondrial dysfunction may alter Wnt signaling and chromatin remodeling in Hq mutant cells (Fig. 1D-F), we assessed the expression of Wnt signaling components and SWI/SNF/BAF complex subunits. Western blots showed higher expression of TCF4/TCF7L2 in *Aifm1* knockdown astrocytes, suggesting an upregulation of Wnt signaling (Fig. 2F). Furthermore, the expression of distinct SWI/SNF/BAF complex subunits, such as the ATPase subunit SMARCA4/BRG1 and the accessory subunit SMARCD3/BAF60C, were significant upregulated in AIF knockdown astrocytes (Fig. 2G). We treated scramble and *siAifm1*-transfected astrocytes either with LPS alone or in combination with BRM104 (2 µM) and assessed changes in cytokine expression via RT-PCR. We found that BRM104 treatment suppressed the expression of *Tnfα*,* Il-1β* and *Il-6* in both LPS-treated control as well as in AIF knockdown cells, with the effect in the latter being more pronounced (Fig. 2H). Consistent with the recently described role of chromatin remodeling (e.g., histone posttranslational modifications) on astrocyte reactivity^60,61^, these data suggest that the SWI/SNF/BAF complex regulates the pro-inflammatory response of astrocytes.

### Complex I deficient iPSC-derived astrocytes negatively affect neurons

**Fig. 3:**
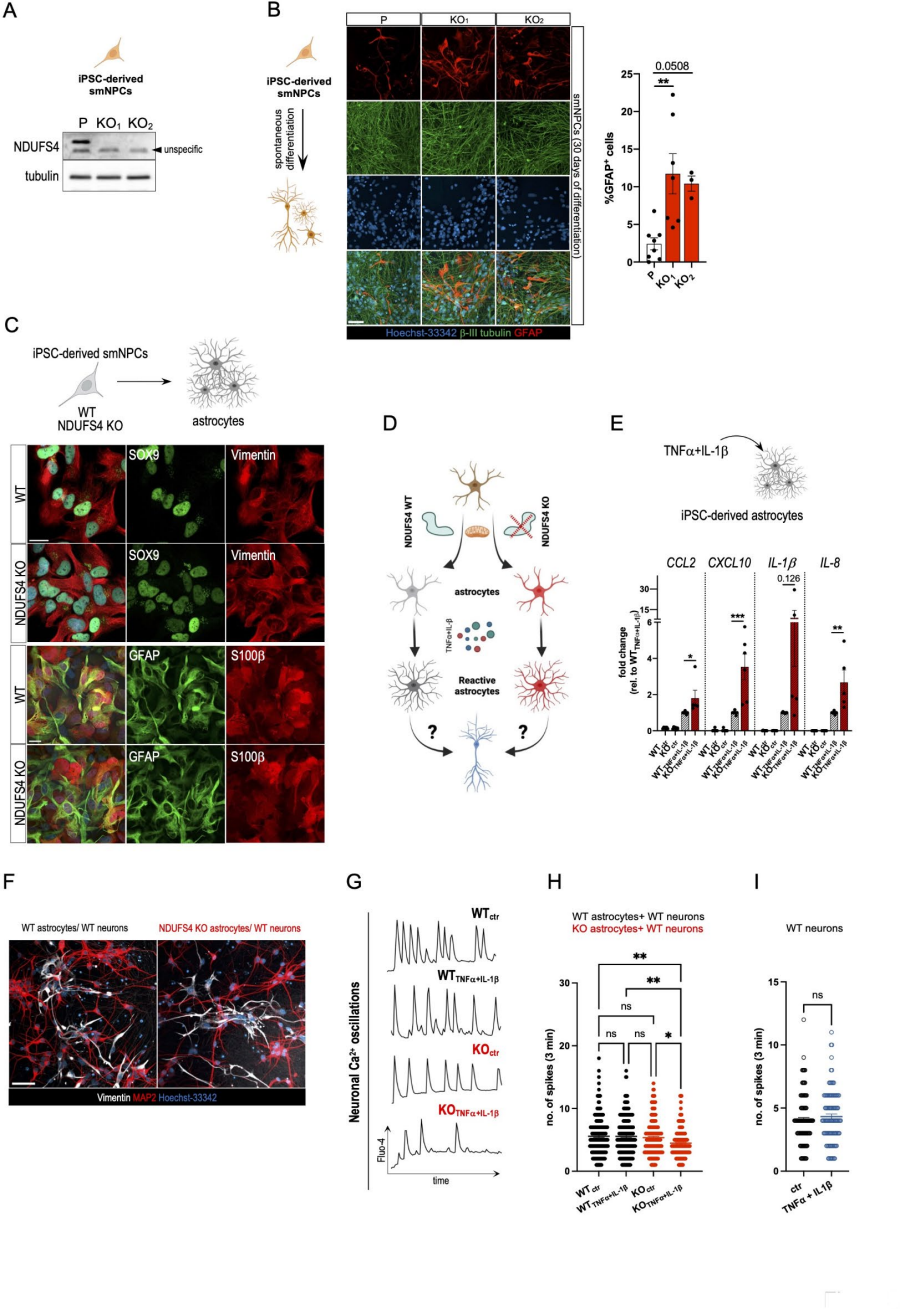
NDUFS4 KO stimulates astrocyte reactivity. (**A**) Cropped immunoblot of NDUFS4 expression levels in iPSC-derived WT and NDUFS4 KO smNPCs (top panel). Tubulin was used as a loading control. (**B**) Representative confocal images of smNPCs following 30 days of spontaneous differentiation (scale bar = 50 μm). Quantification of GFAP^+^ cells in the respective cultures is shown on the right (one-way ANOVA, Bonferroni’s *post hoc* test for multiple comparisons, **p < 0.01; *n* = 3–7). (**C**) Representative confocal images of astrocyte-related markers in WT and NDUFS4 KO smNPC-derived astrocytes (scale bar = 20 μm). (**D**) Schematic representation of the experimental paradigm. (**E**) Gene expression analysis (RT-PCR) of inflammatory cytokines in WT and NDUFS4 KO smNPC-derived astrocytes following 24 h-stimulation with TNFα (50 ng/ml) and IL-1β (50 ng/ml) (one-way ANOVA with subsequent Tuckey’s *post hoc* test for multiple comparisons, *p < 0.05, **p < 0.01, ***p < 0.001; *n* = 3–7). (**F**) Immunofluorescence staining of iPSC-derived neurons co-cultured with either WT or NDUFS4 KO smNPC-derived astrocytes (scale bar = 50 μm). (**G**) Representative Ca^2+^ measurements in iPSC-derived neurons co-cultured with either WT or NDUFS4 KO smNPC-derived astrocytes following 48 h stimulation with TNFα (50 ng/ml) and IL-1β (50 ng/ml). DMSO was used as control (ctr) treatment. Intracellular Ca^2+^ changes were detected using Fluo-4 AM. (**H**) Quantification of spontaneous Ca^2+^ events in iPSC-derived neurons co-cultured with either WT or NDUFS4 KO smNPC-derived astrocytes following 48 h of stimulation with TNFα (50 ng/ml) and IL-1β (50 ng/ml). DMSO was used as control (ctr) treatment (Kruskal-Wallis and Dunn’s multiple comparisons test, ns =  Not significant, *p < 0.05, **p < 0.01, ***p < 0.001; *n* = 3, > 150 cells/condition). (**I**) Quantification of spontaneous Ca^2+^ events in iPSC-derived neurons following 48 h of stimulation with TNFα (50 ng/mL) and IL-1β (50 ng/mL). DMSO was used as control (ctr) treatment (Kruskal-Wallis’ test, ns = not significant; *n* = 2, > 100 cells/condition).

To corroborate our findings in a human cellular model, we employed induced pluripotent stem cell (iPSC)-derived small molecule NPCs (smNPCs) lacking NDUFS4 (Fig. 3A), an accessory subunit of the NADH dehydrogenase module of Complex I^62^ which loss causes Leigh syndrome^35,36,63^. We found that NDUFS4 KO promoted astrogenesis when smNPCs underwent spontaneous differentiation for 30–35 days in vitro (Fig. 3B-C). We subsequently generated smNPC-derived WT and NDUFS4 KO astrocytes via a direct differentiation protocol and confirmed via immunostaining the expression of several astrocytic markers^4^, such as GFAP, S100β, vimentin and SOX9 (Fig. 3C). To assess the impact of defective mitochondrial Complex I on astrocyte reactivity upon pro-inflammatory cytokine treatments (Fig. 3D), we exposed smNPC-derived astrocytes to tumor necrosis factor-α (TNFα, 50 ng/ml) and interleukin-1β (IL-1β, 50 ng/ml) as in previous studies^58,59,64^. We found that TNFα + IL-1β-treated NDUFS4 KO astrocytes had a significantly higher expression of *CCL2*, *CXCL10*,* IL-1β* and *IL-8* compared to WT cells (Fig. 3E). Of note, rotenone treatment did not potentiate the response to TNFα + IL-1β (Supplementary figure S1A-B), suggesting a threshold effect of Complex I inhibition that determines the pro-inflammatory state of astrocytes. Taking advantage of the media compatibility, we seeded iPSC-derived WT or NDUFS4 KO astrocytes on top of differentiated iPSC-derived WT neurons (Fig. 3F). When we performed immunostainings of the co-cultures 48 h after the seeding of the astrocytes, we observed no obvious morphological changes of the neuronal soma as well as of the neuronal network (Fig. 3F). Next, we carried out live-cell Ca^2+^ imaging using Fluo-4 AM to analyze changes in neuronal network activity. In control (DMSO) conditions, we could not detect any difference in spontaneous Ca^2+^ oscillations between neurons that were co-cultured with either WT or NDUFS4 KO astrocytes (Fig. 3G-H). On the contrary, TNFα + IL-1β stimulation led to a significant reduction of spontaneous Ca^2+^ rises in WT neurons co-cultured with NDUFS4 KO astrocytes (Fig. 3G-H). Of note, TNFα + IL-1β stimulation had no effect on neuronal network activity in iPSC-derived neuronal monocultures (Fig. 3I). These data demonstrate that mitochondrial OXPHOS defects may influence the response of astrocytes to an inflammatory stimulation, which in turn can alter neuronal physiology.

## Discussion

In the present study, we report a link between mitochondrial OXPHOS and the SWI/SNF/BAF complex in regulating astrocyte responsiveness to pro-inflammatory stimuli. At least in vitro in human iPSC-derived cells, we provide evidence that Complex I deficient astrocytes negatively influence spontaneous neuronal network activity when exposed to cytokines. Since astrocyte reactivity is a signature commonly observed in brain disorders, our findings reveal that inherited mitochondrial defects can prime inflammatory responses via transcriptionally regulated mechanisms that are potentially druggable.

The SWI/SNF complex is an evolutionarily conserved ATP-dependent chromatin remodeling machinery that regulates the DNA accessibility to the transcriptional machinery^65^. Known also as Brg/Brahma-associated factors (BAF) complex^66^, the SWI/SNF/BAF complex is a multi-subunit assembly that contributes to gene expression regulation. As shown by recent experimental evidence^67–69^, the composition and structural organization of the SWI/SNF/BAF complex determine its mode of action. In this regard, the two mutually exclusive ATPases (BRM/SMARCA2 and BRG1/SMARCA4) bind dedicated core subunits and accessory factors that control the assembly’s deposition and activity on target genes^70^. Importantly, the tightly regulated and cell-type specific expression of SWI/SNF/BAF complex subunits defines the transcriptional profile of the cell in a certain biological context (e.g., development, cell lineage commitment)^66,70^. Besides the well-known role of the SWI/SNF/BAF complex in cancer^71–73^, emerging evidence suggests that inherited SWI/SNF/BAF complex mutations can cause neurodevelopmental disorders^66,74^. Recently, we report that genetic inhibition of the SWI/SNF/BAF complex reduces mitochondrial OXPHOS and promotes NPC differentiation into glia, rather than neurons, in vitro and in vivo^53,56^. By building up on our previous studies on mitochondrial diseases and chromatin remodeling^51–53,56^, we sought to understand how mitochondria could influence NPC differentiation into astrocytes. Pathway analyses of differentially regulated genes in proliferating and differentiating NPCs revealed that altered Wnt signaling and chromatin remodeling are linked to mitochondrial OXPHOS impairment. A closer look at our dataset further showed that different subunits of the SWI/SNF/BAF complex may be differently expressed and recruited to create assembly variants that promote NPC differentiation into astrocytes. Our reasoning is in line with the idea that the composition of the SWI/SNF/BAF complexes determines their biochemical and functional activities, since each SWI/SNF/BAF complex variants create unique chromatin landscapes that define cell lineage commitments in a context- and cell-type specific manner^66,68,75^. Furthermore, we show that pharmacological inhibition of the SWI/SNF/BAF complex attenuates transcriptional pro-inflammatory programs that are triggered in astrocytes upon exposure to cytokines. Our interpretation is that genetic lesions of the mitochondrial OXPHOS system may lower the threshold to which astrocytes become responsive to inflammatory stimuli. The subsequent transcriptional regulation is mediated by SWI/SNF/BAF complex variants with different chromatin remodeling activity to targeted pro-inflammatory genes. Based on our experiments with rotenone-treated astrocytes, we expect that this scenario may not be applicable to all inherited ETC defects, especially for those loss of function mutations that strongly suppress Complex I activity. While our data do not argue about the dependency of astrocytes on mitochondrial bioenergetics for energy production, they strengthen previous evidence that mitochondrial OXPHOS is a key regulator of astrocyte reactivity and immunity^3,19,76–78^.

Recent studies suggest that epigenetic regulators of chromatin remodeling may be valuable targets to tackle reactive astrocytes and therefore fight neurodegenerative diseases and other neurologic disorders, such as multiple sclerosis. In this regard, a phenotypic screening in cultured mouse cortical astrocytes identifies histone deacetylase 3 (HDAC3) as a druggable molecular target to inhibit astrocyte reactivity upon inflammatory challenges (TNFα, IL-1β and complement component C1q)^61^. In support of these conclusions, treatment with a HDAC3 inhibitor ameliorates LPS-induced pathogenic astrocyte reactivity in vivo in mice, resulting in neuroprotection, reduced demyelination of the nerves and diminished oligodendrocyte death in the dorsal column of the spinal cord^61^. Likewise, metabolic rewiring of astrocytes can determine the epigenetic memory of previous inflammatory events by influencing histone acetylation. The subsequent changes in chromatin accessibility defines the engagement of astrocytes to multiple stimulations, with higher production of cytokines sustaining longer CNS inflammation in a mouse model of experimental autoimmune encephalomyelitis^60^. Together, these findings suggest that changes in chromatin remodeling may be an underestimated aspect of astrocyte reactivity and their impact on neurodegenerative processes. Based on that and our experimental evidence, we propose the SWI/SNF/BAF complex could be a new important player in astrocyte-mediated neuroinflammation. We are aware of the limitation of our in vitro models and we look forward to future studies that conclusively can determine the efficacy of SWI/SNF/BAF complex inhibitors in degenerative conditions in which astrocyte proliferation and reactivity have been described as prominent signatures.

## Methods

### Animal work

 Harlequin mutant (JAX stock number: 000501) breeding pairs were obtained from The Jackson Laboratory (Bar Harbor, Maine, USA). Mice were housed in groups of two to four under a 12/12 h light/dark cycle (lights on at 6:00 am) with free access to food (ssniff^®^ V1534-300) and tap water. All experiments were approved and performed in conformity to the guidelines of the State Agency for Nature, Environment and Consumer Protection in North Rhine Westphalia. All procedures complied with ARRIVE guidelines.

### Antibodies

 The following primary antibodies were used: mouse anti-actin (MAB1501, Sigma), rabbit anti-AIF (5318, Cell Signaling) rabbit anti‐BAF47 (8745, Cell Signaling), rabbit anti‐BAF60C (62265, Cell Signaling), rabbit anti‐BAF170 (12760, Cell Signaling), rabbit anti‐BRG1 (ab110641, Abcam), rabbit anti‐BRM (11966, Cell Signaling), rabbit anti‐GAPDH (2118, Cell Signaling), mouse anti‐GFAP (3670, Cell Signaling), chicken anti‐GFAP (ab4674, Abcam), mouse anti‐total OXPHOS (ab110413, Abcam), mouse anti‐MT-CO1 (ab14705, Abcam), rabbit anti‐NDUFB8 (14794‐1‐AP, Proteintech), rabbit anti-NDUFS4 (15849-1-AP, Proteintech), rabbit anti-total β-catenin (8480, Cell Signaling), rabbit anti‐non‐phospho (active) β‐catenin (8814, Cell Signaling), rabbit anti‐S100β (ab52642, Abcam), rabbit anti‐SOX9 (ab185966, Abcam), rabbit anti-STAT3 (12640, Cell Signaling), rabbit anti-phospho-STAT3_Y705_ (9145, Cell Signaling), rabbit anti‐TCF4/TCF7L2 (2569, Cell Signaling), mouse anti‐α tubulin (T6074, Sigma), rabbit anti‐β‐III tubulin (T3952, Sigma), chicken anti-Vimentin (AB5733, Merck Millipore). Alexa Fluor‐conjugated secondary antibodies were obtained from Invitrogen, whereas horseradish peroxidase‐conjugated secondary antibodies were purchased from Promega and Invitrogen.

### Cell culture

 Embryonic neural progenitor cell (eNPC) isolation and culture was performed as described previously^53^. Briefly, eNPCs were isolated from cortices of E12.5-E13.5 Hq mutant and wt mouse embryos. To maintain the genetic identity of subsequent cultures, all embryos were prepped individually. Following dissection, tissues were dissociated with 0.05% trypsin (Gibco) and DNase I (Roche) at 37 °C. The reaction was stopped after 40 min by the addition of NPC proliferation medium (NeuroCult, StemCell) supplemented with 10% FBS (Gibco) and 1% penicillin–streptomycin. Cells were then dissociated via pipetting and filtered through a 70 μm cell strainer. After centrifugation, cell pellets were resuspended in NPC proliferation medium supplemented with EGF (20 ng/ml, Invitrogen), FGF (20 ng/ml, Invitrogen), primocin (InVivoGen), and plasmocin (InVivoGen). eNPCs were then seeded onto laminin (Sigma)‐coated cell culture dishes and the medium was replaced with fresh NPC proliferation medium with half the amount of growth factors and without primocin and plasmocin 48 h after plating. The same medium composition was used for subsequent NPC culture and cells were split when reaching 80% confluency using accutase (Invitrogen). For differentiation experiments, eNPCs were seeded either onto Matrigel‐coated glass cover slips in 12‐well plates (150,000 cells/well), laminin-coated Seahorse microplates (60,000 cells/well) or laminin-coated 6-well plates (300,000 cells/well) in proliferation medium. After 24 h, the medium was replaced by NPC differentiation medium (NeuroCult, StemCell) and cells were then either collected for biochemistry or fixed with 4% PFA at the indicated timepoints.

Cortical astrocytes were isolated from wildtype pups at postnatal day 1–3 as described previously^79^ with slight modifications. Briefly, mouse cortices were dissociated in HBSS supplemented with 0.05% trypsin and DNase I at 37 °C. After 20 min, tissue digestion was stopped by adding DMEM (Gibco) with 10% FBS and 1% penicillin–streptomycin. Cells were further dissociated via pipetting, centrifuged and subsequently transferred into poly-L-lysine (Sigma)-coated T75 flasks in complete medium. After 10–14 days in culture, microglia were erased from astrocyte cultures by l-leucine-methylester (LME, Sigma) treatment. Astrocytes were then cultured for another 1–3 days before being trypsinized and seeded for experiments. For stimulation experiments, astrocytes were treated with LPS (100 ng/ml, supplier) for 24 h in complete medium, supplemented with the BRG1/BRM inhibitor BRM104 (2 µM, MedchemExpress) where indicated.

Human NDUFS4 KO iPSCs (from parental C-14m-s11-NGN2) were generated by Dr. Michael Peitz and Dr. Oliver Brüstle (Universitätsklinikum Bonn, UKB) from an existing clone (C-14 m-s11. For ethics, please see https://hpscreg.eu) as previously described^51^. Cells were cultured under feeder-free conditions in StemMACs iPS‐Brew (Miltenyi Biotec) on vitronectin‐coated 6‐well plates. iPSCs were then differentiated into smNPCs as previously described^80^. Briefly, dissociated iPSC colonies were resuspended in neural induction medium (DMEM‐F12/Neurobasal (Invitrogen), N2 supplement (Invitrogen), B27 supplement without vitamin A (Invitrogen), 2 mM GlutaMAX, 10 µM ROCK inhibitor (Tocris), 10 µM SB‐431542 (Tocris), 1 µM dorsomorphin, 0.5 µM purmorphamine, and 3 µM CHIR99021 (all Miltenyi Biotec) and transferred onto nonadherent plates to induced embryoid body formation. After 3 days, medium was replaced by smNPC maintenance medium (DMEM-F12/Neurobasal, N2, B27, 2 mM GlutaMAX, 0.5 µM purmorphamine, 3 µM CHIR99021 and 150 µM of ascorbic acid) and, on day six, embryoid bodies were triturated via pipetting and plated into Matrigel‐coated 12‐well plates. Thereafter, cells were spilt at a 1:5 to 1:10 ratio using accutase until cultures were free of contaminating non‐smNPCs. For undirected differentiation experiments, smNPCs were seeded onto Matrigel‐coated glass coverslips in 12‐well plates (100,000 cells/well) and, after 48 h, medium was changed to smNPC differentiation medium (DMEM‐F12/Neurobasal, N2, B27, 2 mM GlutaMAX, 1% penicillin-streptomycin, laminin (1:1,000, Sigma)). During the initial week of smNPC differentiation, complete medium changes were performed every 2–3 days, after which only half‐medium changes were conducted. Differentiated smNPCs were then fixed with 4% PFA following 30–35 days of undirected differentiation.

Neuronal differentiation of smNPCs was performed according to a previously published protocol^80^. Briefly, smNPCs were seeded onto Matrigel-coated 6-well plates and smNPC culture medium was changed after 48 h to neuronal induction medium (DMEM-F12/Neurobasal, N2, B27, 2 mM GlutaMAX, 100 ng/ml FGF8 (Peprotech), 1 µM purmorphamine and 200 µM of ascorbic acid). After 8 days, the medium was switched to neuronal maturation medium (DMEM‐F12/Neurobasal, N2, B27, 2 mM GlutaMAX, 10 ng/ml BDNF (Peprotech), 10 ng/ml GDNF (Peprotech), 1 ng/ml TGF-β3 (PeproTech), 200 µM ascorbic acid and 500 µM cAMP) supplemented with 0.5 µM purmorphamine for an additional 2 days. Two days after changing to neuronal maturation medium, cells were dissociated with accutase and seeded onto Matrigel-coated µ-slide 8 well glass bottom plates (ibidi). Experiments were then performed after 3 weeks in maturation medium.

For astrocyte differentiation, confluent smNPC cultures were switched to astrocyte differentiation medium containing DMEM/F-12, GlutaMAX, N2, 1% FBS, 1% penicillin-streptomycin and 20 ng/ml CNTF (PeproTech). Cells were differentiated for at least 6 weeks and split with accutase once confluent. Astrocytes were stimulated with IL-1β (50 ng/ml, PeproTech) and TNFα (50 ng/ml, PeproTech) for 24 h in culture medium supplemented with the BRG1/BRM inhibitor BRM104 (2 µM, MedchemExpress) where indicated. For co-culture experiments, astrocytes were seeded onto neuronal cultures in neuronal medium and allowed to settle for 48 h before drug treatments were started.

### Immunocytochemistry

 PFA-fixed cells were incubated in blocking solution (5% normal goat serum, 0.1% Triton X-100 in PBS) for 1 h at room temperature. Subsequently, primary antibodies were added for 2 h at room temperature or overnight at 4 °C. Cells were then washed in PBST and incubated with appropriate Alexa Fluor-conjugated secondary antibodies (diluted in PBS with 1% BSA) for 1 h at room temperature. Following three washing steps with PBST and Hoechst-33,342 counterstaining, coverslips were mounted onto microscope slides and images were acquired using a LSM900 confocal microscope.

### Intracellular Ca^2+^imaging

 For Ca^2+^ imaging of spontaneous neuronal activity, cells grown in ibidi plates were loaded with 2 µM Fluo-4 AM (Molecular Probes) for 45 min at 37 °C in artificial cerebral-spinal solution (CSS-5: 120 mM NaCl, 5 mM KCl, 1.8 mM CaCl_2_, 15 mM glucose, 25 mM HEPES) supplemented with 10 mM glycine. Thereafter, cells were washed once and incubated for an additional 15 min in CSS-5 without Fluo-4 AM before being imaged. Live cell imaging was performed with an epifluorescence microscope (Zeiss) using a 20x objective with images being taken every 2 s for a total of 3 min. Changes in fluorescence intensities were then quantified in individual cells and spikes counted manually by an experimenter blinded to treatment conditions.

### RNA extraction and RT-PCR

 RNA from cell pellets was extracted using an RNA extraction kit (QIAGEN) and 50–100 ng of mRNA was retrotranscribed into cDNA with qScript cDNA SuperMix (Quanta Biosciences). RT-PCR reactions were carried out with Fast SYBR Green Master Mix on a StepOne Plus Thermocycler (Applied Biosystems). The following primer pairs were used:

Mouse *actin* Fw CTAAGGCCAACCGTGAAAAG.

Rev ACCAGAGGCATACAGGGACA.

Mouse *Il-1β* Fw TGGCAACTGTTCCTG.

Rev GGAAGCAGCCCTTCATCTTT.

Mouse *IL-6* Fw CACTTCACAAGTCGGAGGCT.

Rev TGTGACTCCAGCTTATCTCTTGG.

Mouse *Tnfα* Fw TCCCAGGTTTCTCTTCAAGGGA.

Rev GGTGAGGAGCACGTAGTCGG.

Human *CCL2* Fw GGTTGAGTTTAAGCCAATCCA.

Rev GTGACTGGGGCATTGATTG.

Human *CXCL10* Fw GGTTGAGTTTAAGCCAATCCA.

Rev GGTTGAGTTTAAGCCAATCCA.

Human *IL-1β* Fw CCACAGACCTTCCAGGAGAATG.

Rev GTGCAGTTCAGTGATCGTACAGG.

Human *IL-8* Fw AAGGTGCAGTTTTGCCAAGG.

Rev GTGTGGTCCACTCTCAATCACT.

Human *GAPDH* Fw AGCCACATCGCTCAGACAC.

Rev GCCCAATACGACCAATCC.

### Small interfering RNA

 Astrocytes were transfected with synthetic siRNAs using Lipofectamine RNAiMax (Thermo Fisher) according to the manufacturer’s instructions. The following siRNAs were used: scramble (#AM4611, Thermo Fisher), *siAifm1* (#4390815, ID: s77394, Thermo Fisher). siRNA-treated astrocytes were used for experiments 48 h after transfection.

### RNA sequencing

 RNA was extracted from wt and Hq mutant eNPCs collected at the indicated time points. Approximately 100 ng of extracted RNA were used as input for RNA-Seq library preparation according to the TruSeq Stranded mRNA kit (Illumina). Next generation sequencing libraries were quantified via Qubit HS dsDNA assay (Invitrogen) and library size distribution was determined using a HS D1000 assay on a Tapestation4200 instrument (Agilent). Libraries were equimolarly pooled, clustered at 1200 pM concentration, and sequenced SR 75 cycles on a NovaSeq6000 instrument (Illumina) using an S1 v1.5 chemistry. Samples were demultiplexed and base call files were converted into Fastq format using bcl2fastq2 v2.20 software (Illumina) before alignment to the mouse genome mm10 build GRCm38 using kallisto aligner (v.0,440). Data analysis was performed using Shiny‐Seq ^81^, whereas gene and pathway enrichment analysis was performed with an online gene ontology software (www.geneontology.org).

### Scratch wound assay

 Astrocytes were seeded onto poly-L-lysine-coated 24-well plates and scratch wounds were introduced into confluent cultures by pulling a 200 µml pipette tip through the cell layer (one horizontal and one vertical scratch). Cells were then monitored via live-cell imaging using the IncuCyte (Sartorius) for a total of 24 h. Images were acquired every 2 h and analysis of scratch wound closure was done using ImageJ.

### Statistics

 Data were obtained from at least 3 independent experiments and are represented as mean±SEM. They were statistically analyzed using the Graph Pad Prism software. All statistical tests used as well as the exact number of biological replicates included in each experiment is stated in the Figure legends. The statistical significance was defined as *p* < 0.05 and levels of significance are indicated as *p < 0.05, **p < 0.01, ***p < 0.001, ****p < 0.0001.

### Western blotting

 Proteins from cell pellets or frozen tissue samples were extracted through lysis and sonication in ice-cold RIPA buffer (Sigma), supplemented with protease and phosphatase inhibitors (Roche). Protein lysates were then separated on 8–12% SDS-PAGE gels, followed by transfer onto nitrocellulose membranes (Bio-Rad). Thereafter, membranes were blocked in 5% milk or BSA and incubated with primary antibodies overnight at 4 °C. Following incubation with HRP-conjugated secondary antibodies, immunoblots were imaged using a ChemiDoc Imager (Bio-Rad) and band intensities were quantified with ImageJ.

## Electronic supplementary material

Below is the link to the electronic supplementary material.

Supplementary Material 1.

Supplementary Material 2.

Supplementary Material 3.

Supplementary Material 4.

Supplementary Material 6

## Data Availability

The datasets generated and/or analysed during the current study are available in the NCBI repository (GEO accession number GSE270108).
