# Supplementary material for: Mitochondrial complex I inhibition enhances astrocyte responsiveness to pro-inflammatory stimuli
Source: Sci Rep. 2024 Nov 8;14:27182. doi: 10.1038/s41598-024-78434-y (PMC11549212; doi:10.1038/s41598-024-78434-y)
Supplement: Supplementary file 3 — Supplementary Material 3. [file 41598_2024_78434_MOESM3_ESM.pptx]

## Slide 1
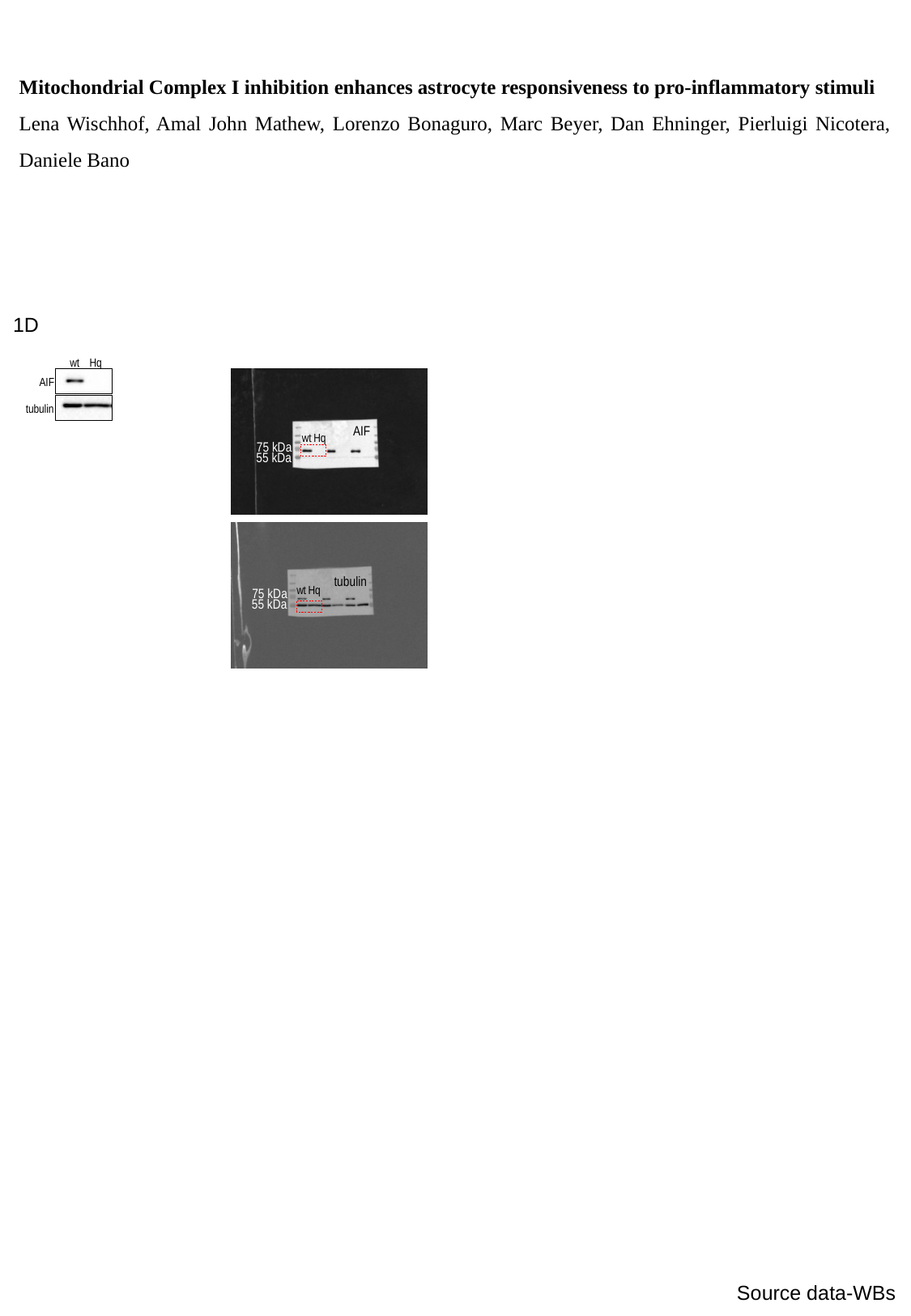

Mitochondrial Complex I inhibition enhances astrocyte responsiveness to pro-inflammatory stimuli
Lena Wischhof, Amal John Mathew, Lorenzo Bonaguro, Marc Beyer, Dan Ehninger, Pierluigi Nicotera, Daniele Bano
1D
Hq
wt
AIF
tubulin
AIF
75 kDa
55 kDa
wt
Hq
tubulin
75 kDa
55 kDa
wt
Hq
Source data-WBs

## Slide 2
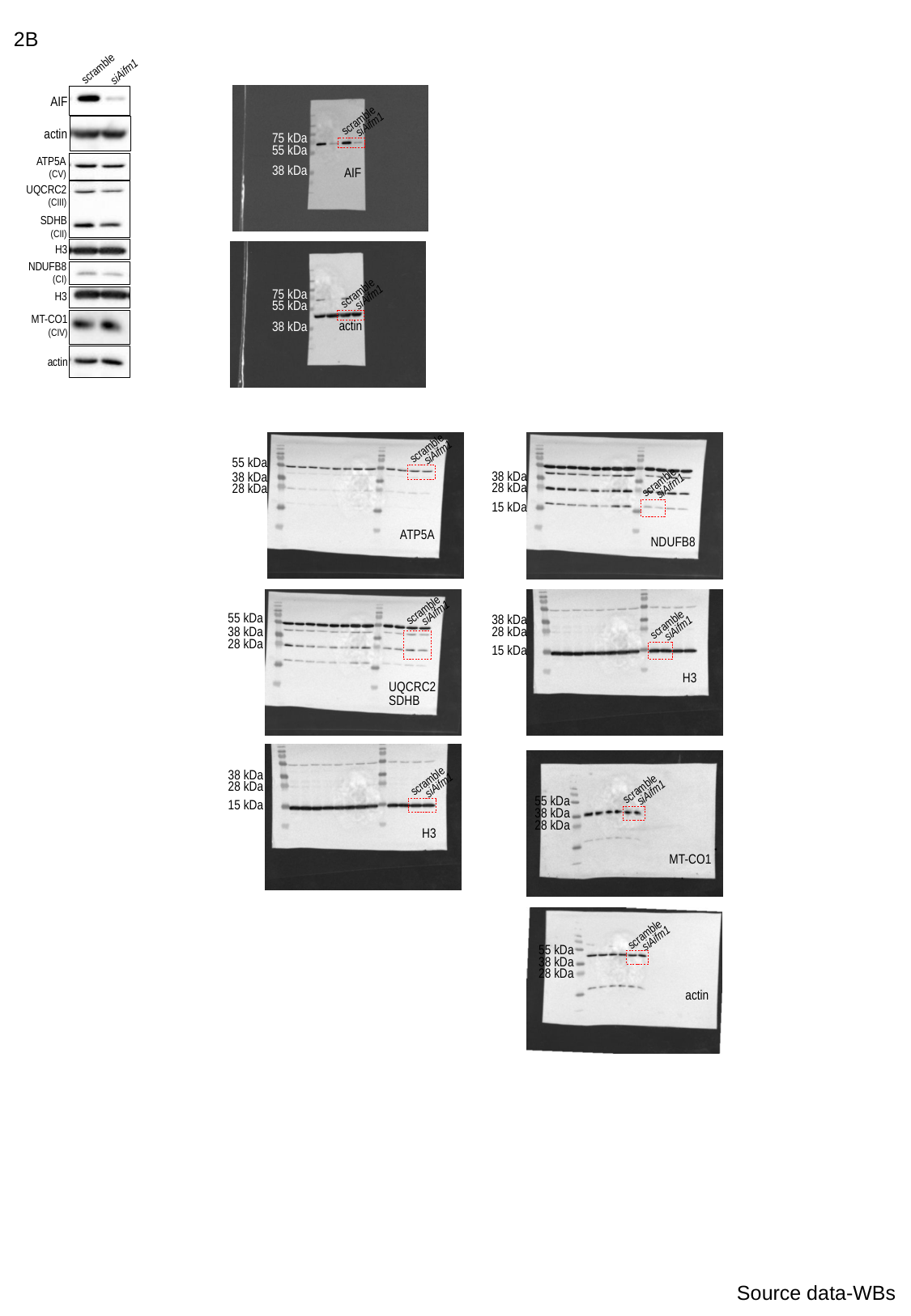

2B
scramble
siAifm1
AIF
actin
ATP5A
(CV)
UQCRC2
(CIII)
SDHB
(CII)
H3
NDUFB8
(CI)
H3
MT-CO1
(CIV)
actin
scramble
siAifm1
75 kDa
55 kDa
38 kDa
AIF
75 kDa
scramble
siAifm1
55 kDa
actin
38 kDa
scramble
siAifm1
55 kDa
38 kDa
28 kDa
ATP5A
38 kDa
scramble
siAifm1
28 kDa
15 kDa
NDUFB8
siAifm1
55 kDa
38 kDa
28 kDa
UQCRC2
SDHB
38 kDa
scramble
siAifm1
28 kDa
15 kDa
H3
scramble
scramble
38 kDa
scramble
siAifm1
28 kDa
15 kDa
H3
scramble
siAifm1
55 kDa
38 kDa
28 kDa
MT-CO1
scramble
siAifm1
55 kDa
38 kDa
28 kDa
actin
Source data-WBs

## Slide 3
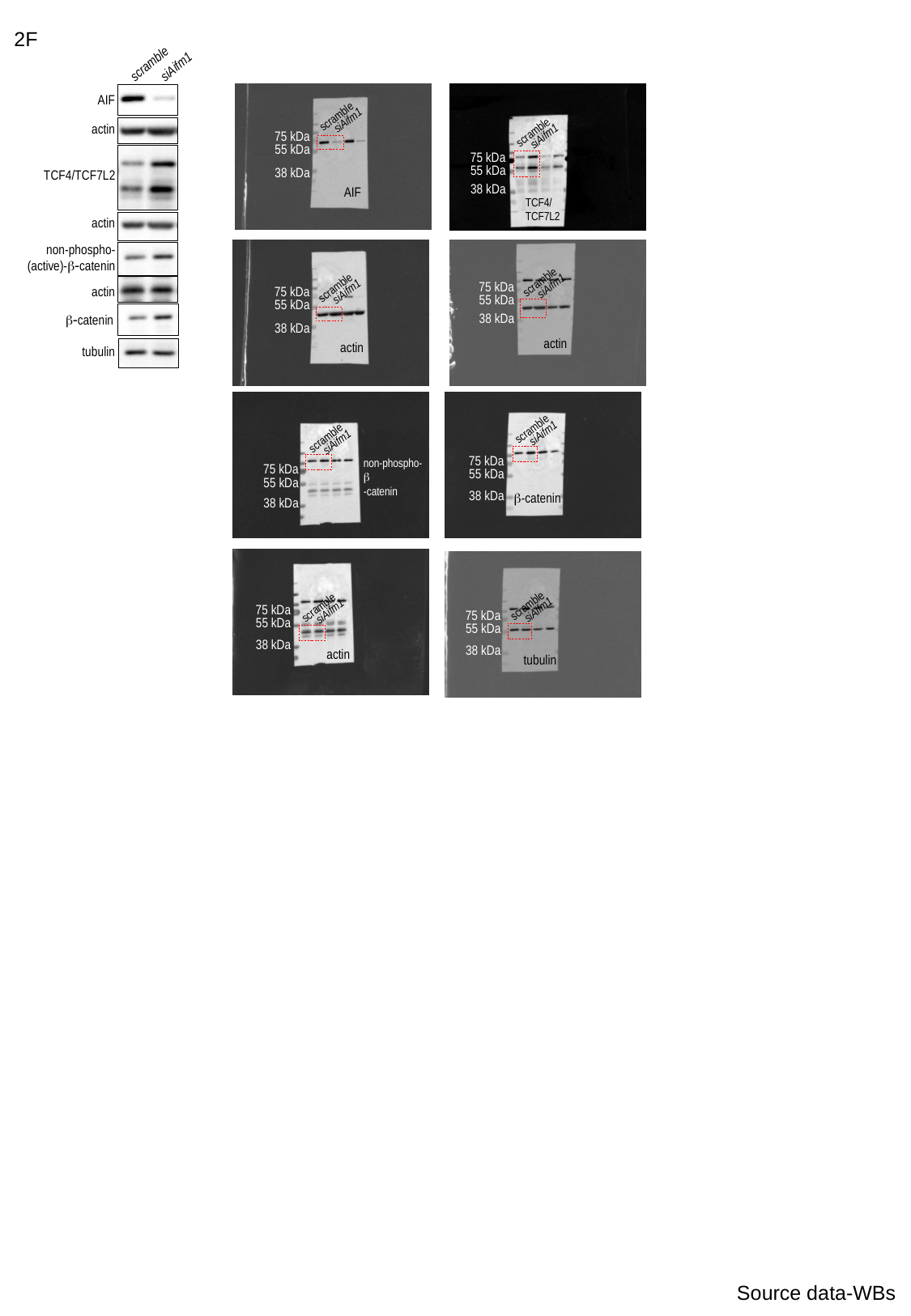

2F
scramble
siAifm1
AIF
actin
TCF4/TCF7L2
actin
non-phospho-(active)-b-catenin
actin
b-catenin
tubulin
scramble
siAifm1
75 kDa
55 kDa
38 kDa
AIF
scramble
siAifm1
75 kDa
55 kDa
38 kDa
TCF4/ TCF7L2
scramble
75 kDa
siAifm1
55 kDa
38 kDa
actin
scramble
75 kDa
siAifm1
55 kDa
38 kDa
actin
scramble
siAifm1
75 kDa
55 kDa
38 kDa
b-catenin
scramble
siAifm1
75 kDa
55 kDa
38 kDa
tubulin
scramble
siAifm1
non-phospho-b
-catenin
75 kDa
55 kDa
38 kDa
scramble
75 kDa
siAifm1
55 kDa
38 kDa
actin
Source data-WBs

## Slide 4
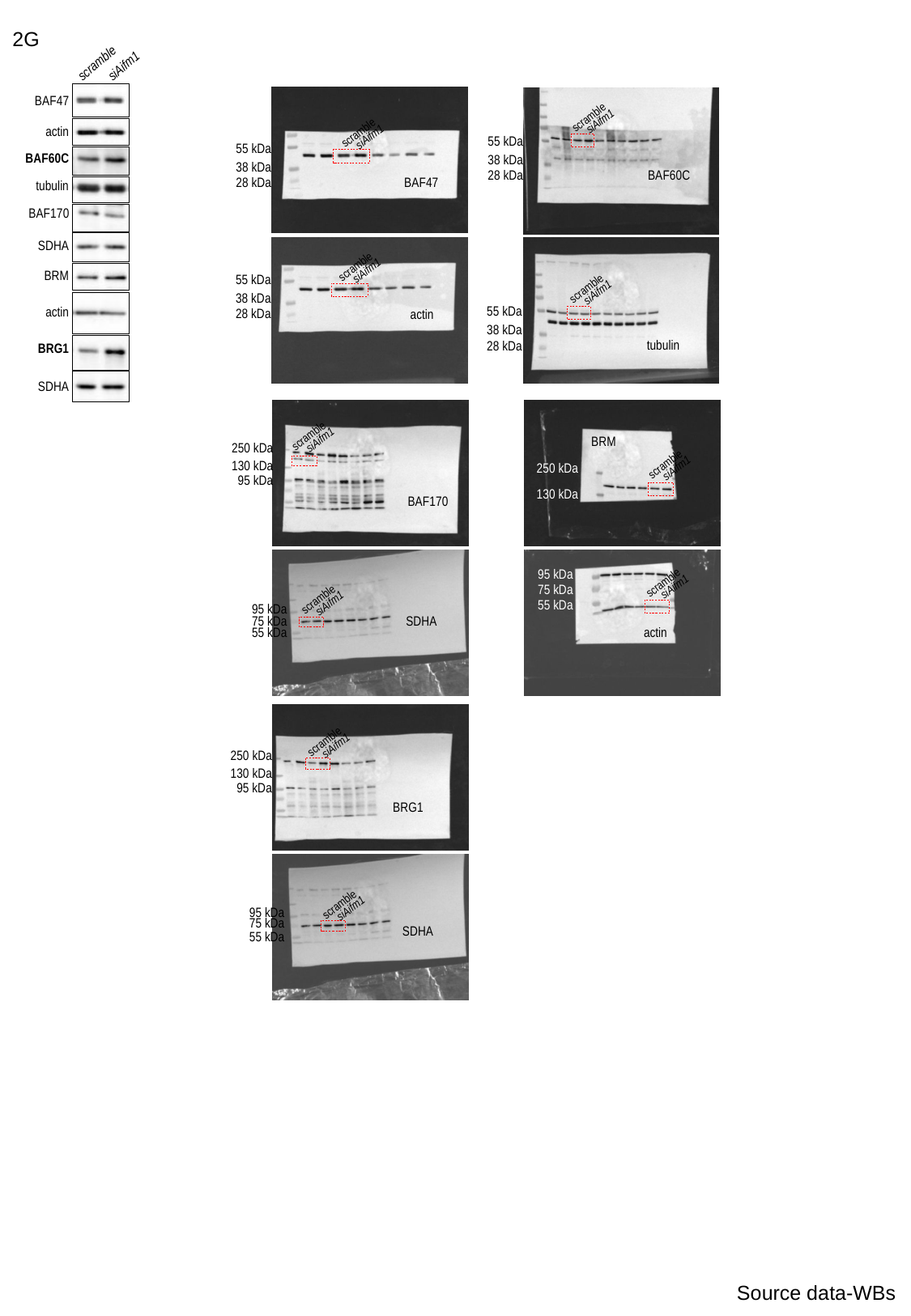

2G
scramble
siAifm1
BAF47
actin
BAF60C
tubulin
BAF170
SDHA
BRM
actin
BRG1
SDHA
scramble
siAifm1
55 kDa
38 kDa
BAF47
28 kDa
scramble
siAifm1
55 kDa
38 kDa
28 kDa
BAF60C
scramble
siAifm1
55 kDa
38 kDa
tubulin
28 kDa
scramble
siAifm1
55 kDa
38 kDa
28 kDa
actin
scramble
siAifm1
250 kDa
130 kDa
95 kDa
BAF170
BRM
scramble
250 kDa
siAifm1
130 kDa
scramble
siAifm1
95 kDa
75 kDa
SDHA
55 kDa
95 kDa
scramble
siAifm1
75 kDa
55 kDa
actin
scramble
siAifm1
250 kDa
130 kDa
95 kDa
BRG1
scramble
siAifm1
95 kDa
75 kDa
SDHA
55 kDa
Source data-WBs

## Slide 5
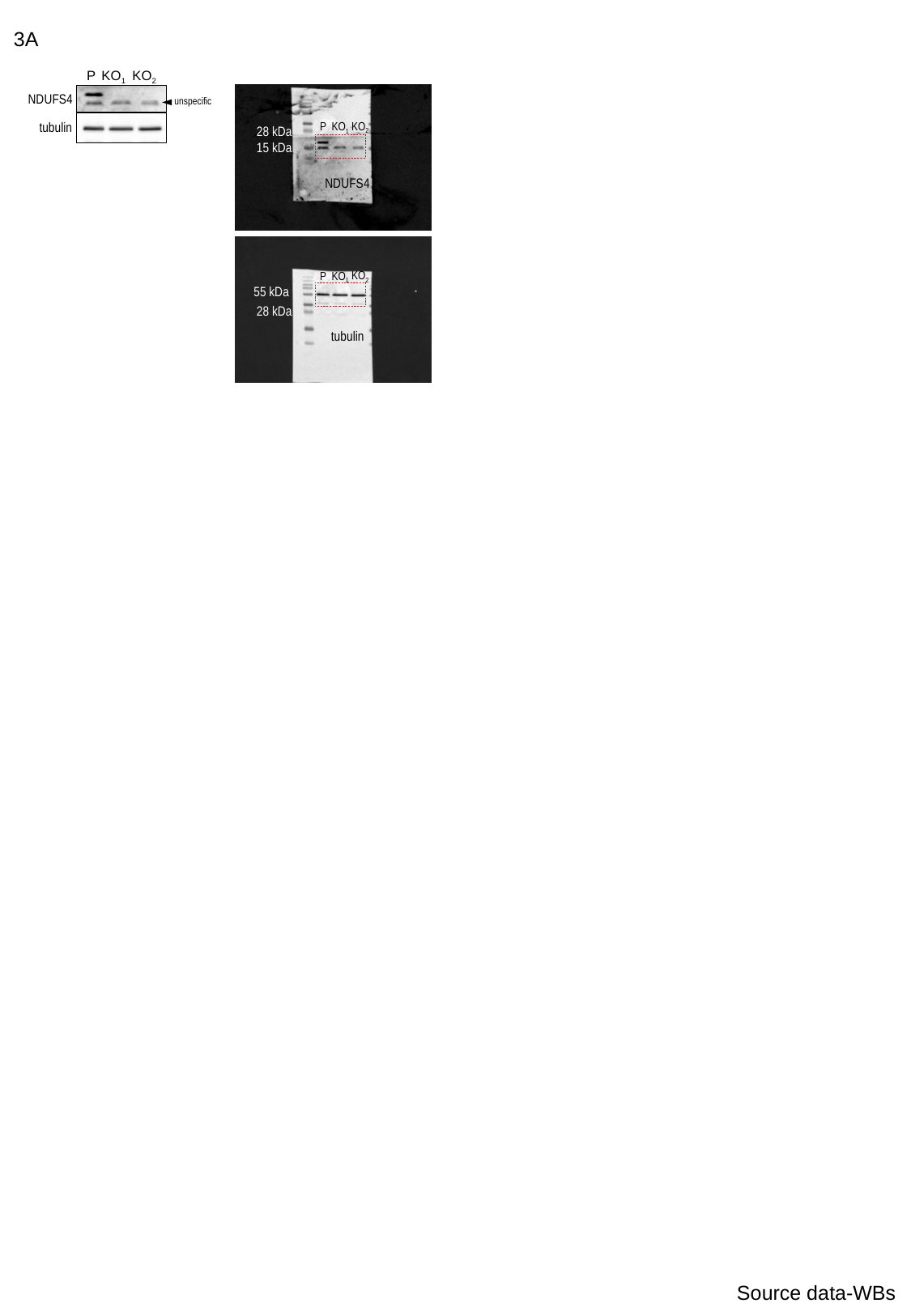

3A
P
KO1
KO2
NDUFS4
tubulin
unspecific
KO2
P
KO1
28 kDa
15 kDa
NDUFS4
KO2
P
KO1
55 kDa
28 kDa
tubulin
Source data-WBs
