# Supplementary material for: Mitochondrial complex I inhibition enhances astrocyte responsiveness to pro-inflammatory stimuli
Source: Sci Rep. 2024 Nov 8;14:27182. doi: 10.1038/s41598-024-78434-y (PMC11549212; doi:10.1038/s41598-024-78434-y)
Supplement: Supplementary file 4 — Supplementary Material 4. [file 41598_2024_78434_MOESM4_ESM.docx]

**Supplementary figure S1 (relative to figure 3). Rotenone treatment does not influence astrocyte response to pro-inflammatory stimuli.** (A) Parental iPSC-derived astrocytes were treated with rotenone (50 or 100 nM) for 24 hours before assessing mitochondrial function using a Seahorse XFE24 analyzer. OCR changes were measured upon sequential treatment with oligomycin, FCCP and rotenone/antimycin. Raw OCR values were normalized to total protein content measured from cell lysates. (B) Parental iPSC-derived astrocytes were treated with rotenone (50 nM) for 24 hours and then stimulated with TNFα (50 ng/ml) and IL-1β (50 ng/ml) for another 24 hours. RT-PCR was used to quantify *IL-1β* , *IL-8*, *CCL2* and *CXCL10* mRNA levels. Data report the mean±SEM (one-way ANOVA; n=3).
