# Supplementary figures and images for: Mitochondrial complex I inhibition enhances astrocyte responsiveness to pro-inflammatory stimuli
Source: Sci Rep. 2024 Nov 8;14:27182. doi: 10.1038/s41598-024-78434-y (PMC11549212; doi:10.1038/s41598-024-78434-y)

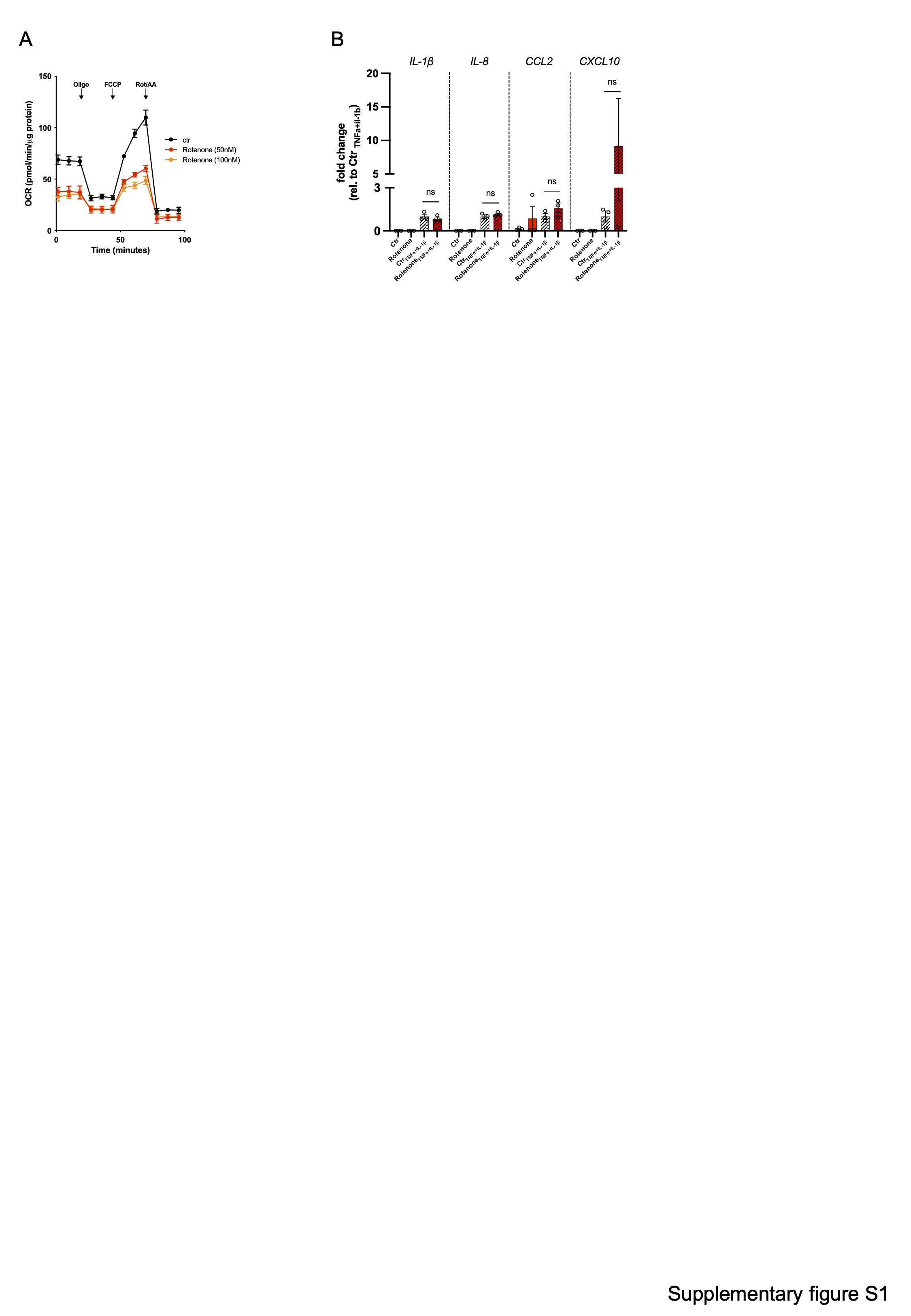

Supplement: Supplementary file 5 — Supplementary Material 6 [file 41598_2024_78434_MOESM5_ESM.jpg]
